# The Cytokine Mediated Molecular Pathophysiology of Psoriasis and Its Clinical Implications

**DOI:** 10.3390/ijms222312793

**Published:** 2021-11-26

**Authors:** Rohan Singh, Sindhuja Koppu, Patrick O. Perche, Steven R. Feldman

**Affiliations:** 1 Center for Dermatology Research, Department of Dermatology, Wake Forest School of Medicine, Winston-Salem, NC 27101, USA; rohsingh@wakehealth.edu (R.S.); koppuss@vcu.edu (S.K.); patrickperche@ufl.edu (P.O.P.); 2 Department of Pathology, Wake Forest School of Medicine, Winston-Salem, NC 27101, USA; 3 Department of Social Sciences & Health Policy, Wake Forest School of Medicine, Winston-Salem, NC 27101, USA

**Keywords:** biologics, IL-17 inhibitors, IL-12/23 inhibitors, IL-23/39 inhibitors, IL-356RN, JAK inhibitors, plaque psoriasis, pustular psoriasis, TNF-α inhibitors

## Abstract

Psoriasis is the result of uncontrolled keratinocyte proliferation, and its pathogenesis involves the dysregulation of the immune system. The interplay among cytokines released by dendritic, T_h_1, T_h_2, and T_h_17 cells leads to the phenotypical manifestations seen in psoriasis. Biological therapies target the cytokine-mediated pathogenesis of psoriasis and have improved patient quality of life. This review will describe the underlying molecular pathophysiology and biologics used to treat psoriasis. A review of the literature was conducted using the PubMed and Google Scholar repositories to investigate the molecular pathogenesis, clinical presentation, and current therapeutics in psoriasis. Plaque psoriasis’, the most prevalent subtype of psoriasis, pathogenesis primarily involves cytokines TNF-α, IL-17, and IL-23. Pustular psoriasis’, an uncommon variant, pathogenesis involves a mutation in IL-36RN. Currently, biological therapeutics targeted at TNF-α, IL-12/IL-23, IL-17, and IL-23/IL-39 are approved for the treatment of moderate to severe psoriasis. More studies need to be performed to elucidate the precise molecular pathology and assess efficacy between biological therapies for psoriasis. Psoriasis is a heterogenous, chronic, systemic inflammatory disease that presents in the skin with multiple types. Recognizing and understanding the underlying molecular pathways and biological therapeutics to treat psoriasis is important in treating this common disease.

## 1. Introduction

Psoriasis is a chronic, systemic inflammatory skin disease that affects approximately 3% or 7.5 million people in the United States [1,2,3]. Worldwide, psoriasis has a prevalence of approximately 1.99% in East Asia, 1.92% in Western Europe, and 1.10% in high-income Southern Latin American countries [4]. Psoriasis occurs equally in both males and females and has a bimodal age of peak occurrence: between 20–30 and 60–70 [5,6]. The underlying pathogenesis of psoriasis results from an interplay between various genetic and environmental factors. Over 80 *human leukocyte genes* (*HLA*) are associated with psoriasis, and HLA-C*06:02 has the strongest association [2]. Environmental factors that can trigger psoriasis include trauma, drugs, infections, smoking, alcohol, and stress. In particular, certain drugs that can exacerbate psoriasis include antimalarials, bupropion, beta-blockers, calcium channel blockers, captopril, and fluoxetine [7]. Scratching, and other forms of trauma, are also associated with the onset of psoriasis, known formally as Koebner’s phenomenon [3].

Psoriasis can be categorized into various subtypes based on clinical presentation and include psoriasis vulgaris or plaque psoriasis, inverse or flexural psoriasis, guttate psoriasis, pustular psoriasis, and erythrodermic psoriasis [1,2]. While psoriasis presents with cutaneous manifestations, it is also associated with other comorbidities. Systemic manifestations of psoriasis include psoriatic arthritis in the small and large joints, affecting 30% of patients with psoriasis, cardiovascular disease, such as myocardial infarctions, diabetes, depression, anxiety, certain malignancies, and obesity [1,8]. 

Over the past two decades, advances in the understanding of the underlying inflammatory molecular pathway in psoriasis have altered management [9]. Specifically, psoriasis pathogenesis is defined by a complex interplay between various cell types and inflammatory cytokines [10]. Both the innate and adaptive immune systems are responsible for psoriatic inflammation. Antimicrobial peptides (AMPs), such as LL-37, β-defensins, and S100 proteins, which are released secondary to keratinocyte stress (i.e., physical injury) can trigger and maintain the inflammatory pathway in psoriasis [11]. Particularly, LL-37 can complex with DNA and RNA to initiate psoriatic inflammation by stimulating plasmacytoid dendritic cells (pDCs) [11]. The maintenance phase of psoriasis is primarily mediated by the adaptive immune system through the TNF-α/IL-23/IL-17 axis. IL-17 can contribute to the maintenance of psoriatic inflammation through an ACT1 dependent and independent pathway. Although IL-17 is traditionally thought to be produced via IL-23 mediated induction of Th17 cells, the innate immune system may also contribute to the production of IL-17 independent of IL-23 [12]. Ultimately, an increased understanding has allowed for the development of biologics and small molecule inhibitors that selectively target psoriasis’ pathogenesis, increasing treatment efficacy while minimizing side effects [10]. Biologics, such as TNF-α inhibitors, IL-12/IL-23 inhibitors, IL-17 inhibitors, and IL-23/IL-39 inhibitors are now considered first-line therapy in moderate to severe psoriasis [2]. JAK inhibitors are also an emerging class of small molecule inhibitors that offer another promising treatment alternative [2]. This narrative review will focus on the underlying molecular pathophysiology of psoriasis, its unique clinical presentation, and the efficacy and safety of emerging and FDA-approved targeted therapeutics. The underlying molecular pathophysiology of comorbidities, such as psoriatic arthritis (PsA), and key variant, pustular psoriasis, will also be explored. 

## 2. Results

### 2.1. The Pathophysiology of Plaque Psoriasis

Psoriasis is a chronic systemic inflammatory disease that presents in the skin due to uncontrolled keratinocyte proliferation and differentiation. The overall pathogenesis of psoriasis can be divided into an initiation and maintenance phase [1].

**Figure 1:**
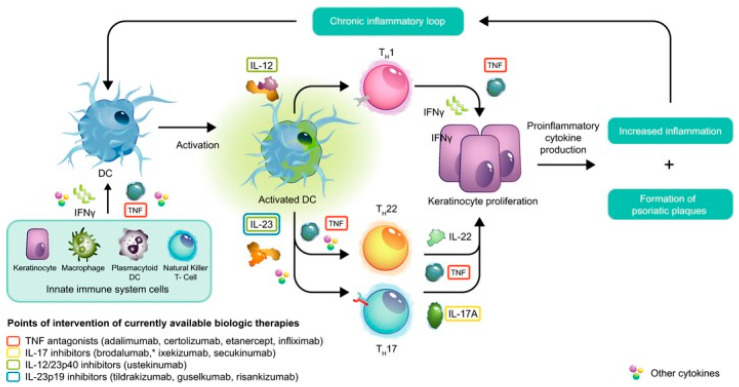
Overview of Psoriasis Molecular Pathogenesis and Targeted Therapies.

The initiation phase is primarily mediated by the effect of AMPs, released by keratinocytes in response to injury, on dendritic cells. The main AMPs involved in the pathogenesis of psoriasis include LL37, β-defensins, and S100 proteins [1]. The effect of LL37 is mediated by its differential binding to either DNA or RNA [1,13]. When bound to DNA, LL37 stimulates toll-like receptor (TLR) 9 to activate pDCs. Activated pDCs produce type I interferons, such as INF-α and IFN-β. These interferons allow myeloid dendritic cells (mDCs) to activate the maturation and differentiation of type 1 helper T cell (T_h_1) and type 17 helper T cells (T_h_17) [1]. T_h_1 cells then produce IFN-γ and TNF-α; and T_h_17 cells produce IL-17, IL-22, and TNF-α. Activated mDCs, in addition to producing type I interferons, can also migrate to lymph nodes and directly secrete various inflammatory cytokines, such as TNF-α, IL-23, and IL-12. IL-23, in turn, facilitates T_h_17 and T_h_22 cell survival and proliferation [1,13]. IL-12 facilitates native T cell differentiation to T_h_1 cell differentiation. TNF-α is a key inflammatory cytokine in psoriasis produced by T_h_1, T_h_17 cells. Activated mDCs can further activate dendritic cells, resulting in an auto-inflammatory loop [1]. When bound to RNA, LL37 stimulates TLR7 and TLR8, which activate pDCs and mDCs, respectively. LL37 bound to RNA can also stimulate slan^+^ monocytes, which secrete TNF-α and IL-23 [1,13]. Β-defensin, another important AMP in psoriasis, acts similarly to LL37 by enhancing self-RNA and self-DNA recognition, activating pDCs [1]. S100 acts to recruit neutrophils, which are commonly present at high levels in psoriatic lesions (Figure 1) [1]. 

The maintenance phase of psoriasis is mediated predominantly by various helper T cell subtypes and their cytokines. The TNF-α/IL-23/IL-17 axis is integral in the underlying pathogenesis of psoriasis (Figure 1) [3]. 

Particularly, the IL-23/IL-17 inflammatory cascade plays a major role in the pathogenesis of psoriasis. IL-23, produced by mDCs, is abundantly found in psoriatic lesions and maintains Th17 cells, a major source of IL-17 [14]. Although IL-17 was believed to be produced in an IL-23 dependent fashion, IL-17 production may occur independently of IL-23 through the innate immune system [12]. Particularly, γδ T cells, group 3 innate lymphoid cells (ILC3s), and natural killer T (NKT) cells are hypothesized to be involved [12]. γδ T cells specifically contain a TCR consisting of an γ and δ chain, contrary to classical T cell α and β chain, and are found in increased numbers in psoriatic skin lesions [12,15]. According to skin analysis, psoriatic lesions contains a high number of γδ T cells capable of producing IL-17 [15], However, according to high-throughput TCR screening, γδ T cells are less than 1% of the total T cells present in psoriatic lesions [16]. ILC3s positive for the natural cytotoxicity receptor (NCR+) are also found in increased numbers in psoriatic lesions. In vitro stimulation of ILC NCR+ produces IL-22, which is implicated in the pathogenesis of psoriasis, and increases the production of S100 AMPs [17,18]. Although the role of NKT cells is not clear, NKT cells are found in psoriatic skin and decrease after treatment of psoriasis [12,19]. Moreover, transplantation of NKT cells leads to the induction of psoriasis, suggesting a contributory role in the pathogenesis of psoriasis [12,20].

IL-17 works via two different mechanisms: one is dependent on the cytoplasmic adaptor protein ACT1 and the other is independent of ACT1 [3].

In the ACT1 adaptor protein-dependent pathway, ACT1 interacts with TNF receptor associated factor (TRAF) 6, which activates transcription factor NF-κB [3]. This initiates the transcription of inflammatory genes and activates p38 mitogen-activated protein kinases, which help stabilize mRNA for cytokines and chemokines involved in psoriasis [3].

The independent pathway acts through the Janus kinase (JAK)-signal transduction and activator of transcription (STAT) pathway [3]. JAKs are non-receptor tyrosine kinases that are involved in cytokine signal transduction. JAK 1, JAK 2, JAK 3, and Tyrosine Kinase 2 (TYK2) are the four members of the JAK family. JAK function is determined by specific cytokine binding [21,22,23]. Cytokine ligand and receptor mediated binding leads to receptor oligomerization and initiation of the underlying molecular pathway [22,23]. This causes the receptor-associated JAK to separate, leading to JAK activation and phosphorylation. An activated JAK can then form a homodimer, by binding with a member of the same family, or heterodimer, by binding a JAK from another family [23]. Dimerization of JAK allows STAT to bind via a docking site, subsequently phosphorylating and activating STAT [1,24]. Activated STAT can dimerize and translocate to the nucleus to regulate transcription, further propagating the inflammatory pathogenesis of psoriasis [14]. 

Specifically, two STAT molecules, STAT1 and STAT3, are crucial in the pathogenesis of psoriasis. STAT1 transduces signals for type I and type II interferons through JAK1 and JAK2 [25]. This allows IFN-γ to sensitize keratinocytes and allows the entry of inflammatory cells into psoriasis lesions. STAT3 promotes the induction and differentiation of T_h_17 cells through different mechanisms [25]. STAT3 can be activated by JAK2/TYK2, induced by IL-23, JAK1/JAK2, and JAK1/TYK2, both induced by IL-6 [25]. Activated STAT molecules, a transcription factor, then translocate to the nucleus to control gene expression, altering transcription to further propagate the inflammatory pathogenesis of psoriasis [25].

#### 2.1.1. Pathogenesis of Pustular Psoriasis

The molecular pathogenesis of pustular psoriasis is similar to the general underlying pathogenesis of psoriasis. However, recently, pustular psoriasis has been associated with mutations in *AP1S3*, *CARD14*, and *IL356RN* [26]. Particularly, the *IL356RN* mutation has been most frequently associated with pustular psoriasis [26]. The *AP1S3* mutation has been found predominantly in patients of European origin, and, in rare cases, in patients of East Asian origin [27,28]. The *CARD14* mutation has also been found primarily in patients with generalized pustular psoriasis and plaque psoriasis [29,30]. However, studies analyzing the *CARD14* and *IL356RN* mutation suggest generalized pustular psoriasis with plaque psoriasis and generalized pustular psoriasis alone have different etiologies [31,32].

The unique mutation in *IL356RN* is specific to pustular psoriasis [33]. Mutated *IL356RN* leads to the disinhibition of IL-1F6, IL-1F8, and IL-1F9. Disinhibition of these cytokines can lead to the activation of proinflammatory pathways, such as NF-κB, activating gene expression of TNF-α, IL-1, IL-6, and IL-8 [33]. These cytokines subsequently control keratinocyte differentiation and proliferation and activate B cell activation and mitogen-activated protein kinase signaling inflammatory pathways [33]. 

Moreover, a gain-of-function mutation of *CARD14* causes overactivation of the NF- κB pathway, which increases expression of pro-inflammatory cytokines IL-1, IL-6, IL-8, and TNF-α, facilitating keratinocyte proliferation and differentiation [31,33,34].

Activated dendritic cells activate and promote the differentiation of T_h_1 cells via IL-12, T_h_22 cells via TNF-α, and T_h_17 cells via IL-23. T_h_1 produces IFN-γ and T_h_17 produces IL-22, TNF-α, and IL-17. TNF-α, IFN-γ, IL-17, and IL-22 lead to keratinocyte proliferation which causes increased inflammation and formation of plaques found in psoriasis. Psoriatic inflammation can establish a chronic inflammatory loop propagating the activation of dendritic cells and subsequent inflammatory processes. 

Abbreviations: DC—dendritic cell; IL—Interleukin; TNF—tumor necrosis factor.

Figure reproduced with permission from Crowley et al. “Safety of selective IL-23p19 inhibitors for the treatment of psoriasis,” the Journal of the European Academy of Dermatology and Venereology, published by John Wiley & Sons Ltd. Hoboken, USA, 2019.

#### 2.1.2. The Pathophysiology of Psoriatic Arthritis

Psoriatic arthritis (PsA), a common comorbidity in patients with psoriasis, affects approximately 15.5% of North American patients, 22.7% of European patients (95% CI: 20.6–25.0%), and 14.0% of Asian patients (95% CI: 117% to 16.3%) with psoriasis [35]. Particularly, PsA is an immune-mediated chronic inflammatory arthropathy that causes inflammation of the entheses and joints, characterized by immune cell infiltration and vascularization [36]. Proinflammatory mediators activate the fibroblast-like synoviocytes, which subsequently invade the adjacent bone and cartilage [36]. Enthesitis is suggested to precede clinical joint involvement and mechanical stress is hypothesized to be a main inciting factor [36,37]. Progression of enthesitis may result in synovitis, leading to the clinical manifestations of PsA [38]. Dactylitis, or inflammation of the entire digit, is a common hallmark of PsA. According to studies utilizing ultrasound imaging, the underlying inflammation is due to tenosynovitis and joint synovitis, while studies using magnetic resonance imaging have also suggested possible enthesitis [39,40]. Molecularly, the pathogenesis of PsA involves cytokines IL-23, IL-17, IL-22, and TNF- α [38]. In animal models, IL-23 stimulates CD3 +, CD4- CD8-, IL-23R +, and ROR γδ resident T cells, which induces production of IL-17, IL-22, and TNF- α. This subsequently promotes osteoproliferation, bone erosion, and inflammation [41,42]. Specifically, CD8 T-cells; CD4+ Th17 cells, responsible for producing IL-17 and IL-22; type 3 innate lymphoid cells, responsible for producing IL-17 and IL-22; and γδ T cells, responsible for producing IL-17 and TNF- α, are implicated in the pathogenesis of psoriasis [37,41,43]. Neutrophils, also recruited by the pro-inflammatory cytokines, lead to the activation of synoviocytes, angiogenesis, and osteoclast-induced bone destruction [37,41]. Specifically, pro-inflammatory cytokine TNF-α stimulates bone resorption, via the RANKL-dependent pathway, by promoting osteoclast differentiation. TNF-α can also induce apoptosis of osteoblasts via inhibition of Wnt signaling, which also subsequently decreases the capacity to repair bone [38]. IL-17 is produced via an IL-23 dependent and independent pathway. Γδ T cells and NKT cells are the primary cells suggested to produce IL-17 independent of IL-23 [38,44]. 

### 2.2. Clinical Manifestations of Psoriasis

#### 2.2.1. Clinical Manifestations of Plaque Psoriasis

Plaque psoriasis is the most prevalent type of psoriasis and comprises approximately 80% of patient cases [2]. It presents as sharply demarcated, erythematous patches or plaques [3,45]. Overlying silver-white scale can be present, and removal can lead to pinpoint bleeding due to increased vascularity in the dermis, known formally as the Auspitz phenomenon [3]. Plaques classically present on the scalp, trunk, gluteal folds, buttocks, and extensor surfaces of extremities, such as elbows and knees [2,45,46]. Psoriasis also commonly affects the nails, with an approximate lifetime incidence of 80–90% [47]. Psoriatic manifestations in the nail can range from the nail bed or matrix to widespread lesions affecting both finger and toe nails [47,48]. Particularly, psoriatic involvement of the nail matrix can lead to red spots in the lunula, pitting, nail plate crumbling, and leukonychia [48]. Nail crumbling, the most severe manifestation of psoriasis in the nails, can also cause nail detachment [48]. Leukonychia appears as white bands, 1 to 2 mm in width [48]. Psoriatic involvement of the nail bed can particularly lead to salmon patches, subungual hyperkeratosis, onycholysis, and splinter hemorrhages [48]. Specifically, onycholysis, or lifting, occurs at the distal or lateral ends of the nails and is typically surrounded by an erythematous border. Salmon patches appear as irregular yellow-orange discolored areas under the nail plate [48]. Splinter hemorrhages are defined as linear red-brown discolored areas of underlying hemorrhage in the nail bed. Subungual hyperkeratosis appears as scales accumulating under the nail plate, with subsequent lifting and thickening of the nail plate [48].

Inverse psoriasis, also known as flexural or intertriginous psoriasis, is a variant of plaque psoriasis [45]. It clinically presents as demarcated, erythematous plaques that typically lack the silver scales present in plaque psoriasis. Inverse psoriasis predominantly involves the skin folds; the most commonly affected areas include the axillary, inframammary, and anogenital folds [45,49]. Other less commonly affected areas include the retroauricular folds, antecubital fossae, popliteal fossae, abdomen, intergluteal cleft, and perineal area [50]. Inverse psoriasis can present in isolation or accompany plaque psoriasis [49].

#### 2.2.2. Clinical Manifestations of Pustular Psoriasis

Pustular psoriasis is the second most common type of psoriasis which presents clinically as sterile pustules [11]. It typically occurs in patients in their 40 s, although cases have been reported in infants and young children. Pustular psoriasis is also more common in females [33]. Pustular psoriasis can present as generalized pustular psoriasis or localized pustular psoriasis, depending on BSA involvement. Particularly, pustular psoriasis may present as overlying sterile pustules on pre-existing psoriatic plaques [33]. Generalized pustular psoriasis is typically characterized by 2 to 3 mm sterile pustules overlying erythematous, painful skin. In the generalized form, the lesions are diffusely distributed, and patients present with malaise, high-grade fever, neutrophilic leukocytosis, peripheral eosinophilia, and elevated C-reactive protein. Additionally, extra-cutaneous manifestations, such as cholangitis, epigastric pain, cholestasis, arthritis, acute renal failure, and interstitial pneumonitis, warrant hospitalization [1,2,33,51].

### 2.3. Biological Therapies for Plaque Psoriasis

Biologics are immunomodulators that specifically target certain cytokines common to the inflammatory pathway of psoriasis. These therapeutics have gained popularity in the treatment of psoriasis and are a mainstay of treatment. Currently, there are 11 FDA-approved biologics to treat psoriasis targeting the important TNF-α/IL-23/IL-17 inflammatory axis [52]. The four main classes are TNF-α inhibitors, IL-12/IL-23 inhibitors, IL-17 inhibitors, and IL-23/IL-39 inhibitors (Figure 1) [2]. 

#### 2.3.1. TNF-α Inhibitors

TNF-α inhibitors are a class of biologics used to treat moderate to severe plaque psoriasis. Specifically, TNF-α is a proinflammatory cytokine implicated in the pathogenesis of psoriasis that activates the NF-κB signaling pathway [53]. There are currently four FDA-approved TNF α inhibitors: etanercept, certolizumab, infliximab, and adalimumab.

Etanercept, certolizumab, infliximab, and adalimumab are all also currently FDA-approved to treat PsA [38]. 

Etanercept is a recombinant fusion protein that inhibits the binding of TNF-α to TNF receptors 1 and 2. Despite Etanercept’s Fc region complement binding domain, it does not have antibody-dependent cellular cytotoxicity [53,54,55,56]. Certolizumab is a PEGylated TNF-α antibody fragment. Particularly, certolizumab does not possess the Fc portion of immunoglobulin, lowering the rate of anti-drug antibodies formation, and thus immunogenicity and loss of efficacy. Certolizumab also does not also bind IgG neonatal Fc receptor, minimizing placental transfer in pregnant women [53,54,55,56,57]. Infliximab is a chimeric monoclonal IgG1 antibody consisting of the human IgG1κ constant region and a high-affinity anti-human TNF-α antibody murine variable antigen-binding region. Specifically, infliximab can bind to both membrane-bound and soluble TNF-α [53,54,55,56]. Adalimumab is a fully human monoclonal IgG1 antibody that can also bind to both membrane-bound and soluble TNF-α (Table 1) (Table 2) [53,54,55,56].

In a multi-center phase IIIb, randomized, double-blind, placebo-controlled clinical trial, evaluated the efficacy of etanercept. More patients treated with etanercept (48.2%) achieved a ≥75% reduction from baseline psoriasis area and severity index (PASI-75) after 16 weeks of treatment compared to placebo (11.9%, *p* < 0.0001) [54]. A phase IV, open-label, single-arm estimation study evaluated the efficacy of etanercept therapy in patients who previously failed adalimumab. Both patients positive for anti-adalimumab antibodies (ADAs) (47.5% (95% CI: 31.5–63.9)) and patients negative for ADAs (50% (95% CI: 27.2–72.8) achieved PASI-75 after 12 weeks of treatment with etanercept [58]. The results supported etanercept as a satisfactory treatment for patients with secondary failure to adalimumab, regardless of ADA status [58].

The efficacy of certolizumab was evaluated in two multi-center phase III randomized, double-blind, placebo-controlled clinical trials (CIMPASI-1 and CIMPASI-2). After 16 weeks of treatment, PASI-75 was achieved in more patients who received certolizumab 400 mg (CIMPASI-1: 75.8%, CIMPASI-2: 82.6%) and certolizumab 200 mg (CIMPASI-1: 66.5%, CIMPASI-2: 81.4%), compared to placebo (CIMPASI-1: 6.5%, CIMPASI-2: 11.6%, all *p* < 0.0001) [59]. The response rate to both doses of certolizumab was maintained through week 48 in both trials. Although a higher proportion of patients achieved PASI-75 in a dose-dependent manner in the CIMPASI-1 trial, there was little difference between doses of certolizumab in the CIMPASI-2 trial [59].

A prospective, randomized controlled trial evaluated the efficacy of infliximab, compared to etanercept in patients with moderate-to-severe plaque psoriasis. More patients treated with infliximab (72%) achieved PASI-75 after 24 weeks of treatment, compared to etanercept (35%, *p* = 0.01) [60]. After 48 weeks of maintenance treatment, the difference in patients who achieved PAS-75 was smaller between patients treated with infliximab (76%), compared to etanercept (50%, *p* = 0.65) [60]. 

Due to the absence of clinical trials comparing efficacy among all TNF-α inhibitors, a network meta-analysis compared the efficacy of TNF-α inhibitors among one another by analyzing the number of reported patients who achieved PASI 90. Ref. [61] The highest efficacy was reported in patients who received infliximab (56.8% [95% CI: 50.4%, 62.9%]), followed by certolizumab 400 mg (49.6% [95% CI: 43.0%, 56.3%]), adalimumab (43.0% [95% CI: 38.7%, 47.4%]), certolizumab 200 mg (42.2% [95% CI: 35.3%, 49.4%]), and etanercept (18.0% [95% CI: 14.5%, 22.2%]) [61].

The safety profiles of TNF-α inhibitors are well described. Common adverse effects of all TNF-α inhibitors include nasopharyngitis, upper respiratory tract infections, and infection site reactions [2,55,56]. A five-year surveillance registry of psoriatic patients treated with etanercept did not identify any new safety signals, consistent with its current safety profile [62]. Additionally, a seven-year interim analysis of an ongoing international prospective observational registry evaluating the long-term safety of adalimumab in psoriatic patients reported no new safety signals, consistent with its current safety profile [63]. A longitudinal, multicenter, disease-based registry (PSOLAR) of 11,466 adult psoriasis patients who received either adalimumab, etanercept, infliximab, ustekinumab, methotrexate/nonbiologics, or non-methotrexate/non-biologics evaluated the risk of serious infection in a real-world patient population [64]. A total of 323 serious infections were reported and 287 prolonged or caused patient hospitalization. The rate of serious infection varied by treatment cohort and were 0.83, 1.47, 1.87, 2.49, 1.5 and 1.28 per 100 patient-years in the ustekinumab, etanercept, adalimumab, infliximab, non-methotrexate/non-biologic, and methotrexate/non-biologic treatment cohorts, respectively. Patients treated with adalimumab and infliximab had a higher risk for serious infection, compared to non-methotrexate/non-biologic treatment cohorts (*p* < 0.001 and *p* = 0.002, respectively) [64]. Further analysis of the PSOLAR registry analyzed the risk of malignancy with TNF-α inhibitor therapy compared to methotrexate and ustekinumab in patients with no history of cancer [65]. Although there was no increased risk for malignancy with methotrexate or ustekinumab treatment, TNF-α inhibitor therapy was associated with an increased risk for malignancy with 12 or more months of treatment, compared to no TNF-α inhibitor therapy (OR: 1.54, *p* = 0.01) [65]. However, when results were adjusted for exposure to individual TNF-α inhibitors, there was no increased risk for malignancy with exposure to either etanercept (OR: 1.37, *p* = 1.007), infliximab (OR: 1.01, *p* = 0.958), or adalimumab (OR: 1.37, *p* = 1.099) [65]. Still, all TNF-α inhibitors have a black box warning due to the risk of serious infection and malignancy. Therefore, a tuberculin (TB) test should be performed in all patients before starting and annually during the course of treatment [2]. If patients have active TB, treatment is required prior to starting treatment with TNF-α inhibitors. The presence of latent TB in patients necessitates prophylactic treatment of TB and a one-month delay before starting TNF- α inhibitor therapy due to the underlying risk of conversion to active disease [2]. Moreover, etanercept is specifically contraindicated in patients with active tuberculosis, hepatitis B, advanced congestive heart failure, demyelinating diseases, and sepsis [55]. Infliximab is specifically contraindicated at higher doses in moderate to severe heart failure [55]. Adalimumab and certolizumab are both contraindicated in patients with active infection, sepsis, and class III or IV congestive heart failure (Table 1) [55].

#### 2.3.2. IL-12/IL-23 Inhibitors

Currently, ustekinumab is the only FDA-approved IL-12/IL-23 inhibitor used to treat moderate to severe plaque psoriasis. Ustekinumab is a monoclonal human IgG1 antibody that targets p40, a subunit of both IL-12 and IL-23. IL-12 and IL-23 are both cytokines secreted by mDCs and facilitates the differentiation of Th1 and Th17 cells. Particularly, ustekinumab inhibits psoriasis pathogenesis through the IL-12/TH1 and IL-23/Th17 pathways (Table 2) (Table 3) [2,53,71,72].

Ustekinumab is also currently FDA-approved to treat PsA [38,70,73].

In a multi-center, phase III, randomized, double-blind placebo-controlled clinical trial in Korean and Taiwanese patients with moderate to severe psoriasis, more patients treated with ustekinumab 45 mg achieved PASI-75 (67.2%) and a PGA of 0 or 1 (70.5%), compared to placebo (5.0% and 8.3%, respectively; *p* < 0.001 for both) [74].

Two replicate, multi-center, phase III randomized, double-blind, placebo and active comparator-controlled trials (UltIMMa-1 and UltIMMa-2), evaluated the efficacy of ustekinumab compared to risankizumab in patients with moderate to severe plaque psoriasis [75]. Primary endpoints measured were patients who achieved PASI-90 and a static physician’s global assessment (sPGA) of 0 or 1 after 16 weeks of treatment. After 16 weeks of treatment, more patients treated with risankizumab achieved PASI 90 in both UltIMMa-1 and UltIMMa-2 (75.3% and 74.8%), compared to ustekinumab and placebo (42.0% and 47.5%; 4.9% and 2.0%, respectively; *p* < 0.0001 for all) [75]. Additionally, after 16 weeks of treatment, more patients treated with risankizumab achieved a sPGA of 0 or 1 in both UltIMMa-1 and UltIMMa-2 (87.8% and 83.7%), compared to ustekinumab and placebo (63.0% and 61.6%; 7.8% and 5.1%, respectively; *p* < 0.0001 for all) [75].

Two replicate, multi-center phase III, randomized, double-blind, placebo and active comparator-controlled trials (AMAGINE-2 and AMAGINE-3), evaluated the efficacy of ustekinumab compared to brodalumab 210 mg in patients with moderate to severe psoriasis [76]. The primary endpoint measured was the number of patients who achieved PASI-100 after 12 weeks of treatment. After 12 weeks of treatment, more patients treated with 210 mg brodalumab achieved PASI-100 in both trials (44% and 37%, respectively), compared to ustekinumab (22% and 19%, respectively; *p* < 0.001 for all) [76]. Another multi-center, phase IIIb randomized, double-blind, head-to-head parallel group trial evaluated the efficacy of ustekinumab compared to secukinumab in patients with moderate to severe psoriasis [77]. Primary endpoints measured included patients who achieved PASI-90 and investigator’s global assessment modified 2011 (IGA mod) scores of 0 to 1. After 52 weeks of treatment, more patients treated with secukinumab 300 mg achieved PASI-90 and an IGA mod of 0 to 1 (73.2% and 76.0%, respectively), compared to ustekinumab 45/90 mg (59.8% and 60.2%, *p* < 0.0001 for both) [77].

According to a network meta-analysis used to compare efficacy of various biologics to treat moderate to severe plaque psoriasis, PASI-90 was achieved in 47.9% (95% CI: 41.4%, 54.2%), 45.7% (95% CI: 41.2%, 50.3%), and 44.6% (95% CI: 39.2%, 49.8%) of patients treated with ustekinumab 90 mg, weight-based dosage, and 45 mg, respectively [61]. 

Ustekinumab is a well-tolerated biologic with a defined adverse effect profile. The most common adverse effects include headache, nasopharyngitis, upper respiratory tract infections, fatigue, and pruritus [2,55,56,71]. The DERMBIO registry of 2161 Danish patients with moderate to severe plaque psoriasis evaluated the safety of ustekinumab (*n* = 1055), compared to TNF-α inhibitors (adalimumab, etanercept, and infliximab) [78]. Ustekinumab had the lowest risk for drug discontinuation and highest drug survival, compared to TNF-α inhibitors. There were 463 total reported adverse events with treatment series. Infection (*n* = 235), neurological (*n* = 25), and cardiovascular (*n* = 10) events were the most commonly reported adverse effects. The safety profile for ustekinumab was consistent with past studies and supported its higher drug survival compared to TNF-α inhibitors [63,78]. Currently, ustekinumab is contraindicated in patients who have active infections and is not associated with any black box warnings (Table 3) [79].

#### 2.3.3. IL-17 Inhibitors

IL-17 inhibitors are another class of biologics used to treat moderate to severe plaque psoriasis. Specifically, IL-17 is a proinflammatory cytokine implicated in the pathogenesis of psoriasis through an ACT1 adaptor protein dependent and independent pathway [3]. There are currently three FDA-approved IL-17 inhibitors: secukinumab, ixekizumab, and brodalumab. Bimekizumab is currently undergoing phase III trials [80,81,82,83].

Secukinumab is a human IgG1κ monoclonal antibody that blocks IL-17A and is associated with low immunogenicity [53,80,81,82,83]. Ixekizumab is a humanized IgG4 monoclonal antibody that also blocks IL-17A, although its binding affinity to IL-17A is greater than secukinumab. Particularly, ixekizumab also lacks the ability to bind complement and has had the hinge region amino acid replaced to prevent the formation of antibodies [53,80,81,82,83]. Brodalumab is a human IgG2 monoclonal antibody that blocks IL-17 receptor type A, thus inhibiting the signaling of IL-17A, C, E, and F [80,81,82]. Bimekizumab is a humanized IgG1 monoclonal antibody that inhibits IL-17A and IL-17F. Specifically, bimekizumab’s affinity to IL-17A is greater than IL-17F (Table 2) (Table 4) [53,80,81,82,83,84]. 

Secukinumab and ixekizumab are currently FDA-approved to treat PsA [38]. Brodalumab has completed phase III trials and bimekizumab has been studied in phase II/III trials for PsA [85,86].

In a phase II randomized, double-blind, placebo-controlled study, more patients treated with secukinumab (56.5%) achieved histologic reversal after 12 weeks of treatment, compared to placebo (0%, *p* < 0.001) [80]. In addition, after 12 weeks of treatment, more patients treated with secukinumab (62.5%) achieved PASI 75, compared to placebo (0%, *p* < 0.001) [80]. In a randomized, double-blind, phase IIIb study for the treatment of moderate-to-severe plaque psoriasis secukinumab was compared to ustekinumab, and after 16 weeks of treatment, more patients treated with secukinumab (79.0%) achieved PASI 90 compared to patients treated with ustekinumab (57.6%, *p* < 0.0001) [87]. Secukinumab was compared to ustekinumab in another randomized controlled, phase 3b study for the treatment of moderate-to-severe plaque psoriasis. More patients treated with secukinumab achieved PASI 90 (66.5%) after 12 weeks of treatment compared to ustekinumab (47.9%, *p* < 0.0001). In addition, more patients treated with secukinumab (72.3%) achieved Investigator’s Global Assessment (IGA) 0/1 compared to ustekinumab (55.4%, *p* < 0.0001) [77].

Two prospective, multi-center phase III, double-blind trials (UNCOVER-2 and UNCOVER-3) evaluated the efficacy of ixekizumab compared to etanercept and placebo in patients with moderate to severe psoriasis [82]. After 12 weeks of treatment more patients treated with ixekizumab every 2 weeks and every 4 weeks achieved PASI 75 (UNCOVER-2: 89.7% and 77.5%, UNCOVER-3: 87.3% and 84.2%, respectively), compared to etanercept 50 mg twice weekly (UNCOVER-2: 41.6%, UNCOVER-3: 53.4%, all *p* < 0.0001) [82].

A multi-center phase III, double-blind, placebo-controlled study evaluated the efficacy of brodalumab in patients with moderate to severe psoriasis (AMAGINE-1) [88]. After 12 weeks of treatment, PASI 75 was achieved in more patients treated with brodalumab 210 mg every 2 weeks (83%) and brodalumab 140 mg every 2 weeks (60%), compared to placebo (3%, *p* < 0.001 for both) [88]. 

A multi-center, phase III, randomized, double-blind, placebo-controlled trial (BE READY) evaluated the efficacy of bimekizumab in patients with moderate to severe plaque psoriasis. After 16 weeks of treatment, more patients treated with bimekizumab 320 mg every 4 weeks achieved PASI 90 and an IGA score of 0 or 1 (91% and 93%, respectively), compared to placebo (1% and 1%, *p* < 0.0001 for both) [83]. A multi-center, phase III, randomized, double-blind, active comparator and placebo-controlled trial (BE VIVID) evaluated the efficacy of bimekizumab compared to ustekinumab and placebo in patients with moderate to severe psoriasis. After 16 weeks of treatment, more patients treated with bimekizumab achieved PASI 90 (85%) compared to ustekinumab (50%, *p* < 0.0001) [89]. A multi-center, phase III, double-blind trial also evaluated the efficacy of bimekizumab compared to adalimumab in patients with moderate to severe plaque psoriasis. After 16 weeks of treatment, more patients treated with bimekizumab (86.2%) achieved PASI 90 compared to adalimumab (47.2%), *p* < 0.001) [90]. A multi-center, phase IIIb, randomized, double-blind, active comparator-controlled parallel group trial evaluated the efficacy of bimekizumab, compared to secukinumab, in patients with moderate-to-severe plaque psoriasis. After 16 weeks of treatment, more patients treated with bimekizumab (61.7%) achieved PASI 100 compared to secukinumab (48.9%, *p* < 0.001) [91].

Two network meta-analyses compared the efficacy among all IL-17 inhibitors. A 2019 meta-analysis analyzing randomized control trials measured efficacy by percent of patients achieving PASI 100 and then ranked treatments from high to low according to their surface under the cumulant ranking curves (SUCRA): brodalumab 210 mg (85%), ixekizumab 80 mg/2 weeks (83%), ixekizumab 80 mg/4 weeks (77%), brodalumab 140 mg (63%), secunkinumab 300 mg (62.4%), and secukinumab 150 mg (31%) [92]. Another 2021 network meta-analysis compared the efficacy, measured by percent of patients who achieved PASI 90, among all IL-17 inhibitors in psoriasis. The highest efficacy was reported in patients treated with brodalumab (78.8% [95% CI: 74.0 83.0%]), ixekizumab (72.1% [95% CI: 62.7%, 80.1%]), and secukinumab (67.0% [95% CI: 62.8%, 71.0%]) [61].

The safety profiles of IL-17 inhibitors are well described and are mild to moderate. Common adverse effects of all IL-17 inhibitors include upper respiratory tract infections and injection site reactions. Mucocutaneous candidiasis has also been reported with IL-17 inhibitor therapy [55,80,81]. A 2019 meta-analysis analyzing randomized control trials measured safety profiles of all IL-17 inhibitors from high to low based on short-term risk for adverse events according to their SUCRA: ixekizumab 80 mg/4 weeks (4.5%), ixekizumab 80 mg/2 weeks (7.5%), secukinumab 150 mg (22.7%), brodalumab 210 mg (23.7%), secunkinumab 300 mg (33.9%), brodalumab 140 mg (38.1%) [92]. Secukinumab is contraindicated in patients undergoing PUVA sessions or in patients with premalignancy, demyelinating disease, optic neuritis, multiple sclerosis, congestive heart failure, fever, jaundice, and markedly elevated liver enzymes, and ixekizumab is contraindicated in patients who have a hypersensitivity to the biology. Both of these biologics are not currently associated with any black box warnings. [55,93,94]. Brodalumab is associated with a black box warning due to the risk of suicidal ideation and behavior [55,95]. Brodalumab is contraindicated in patients with Crohn’s disease and those with non-resolving infections (Table 4) [55,95].

#### 2.3.4. IL-23/IL-39 Inhibitors

IL-23/IL-39 inhibitors are a class of biologics used to treat moderate to severe plaque psoriasis. IL-39, a heterodimer composed of IL-23p19 and Epstein-Barr virus-induced gene 3, is a new member of the IL-12 family and is targeted by p19 inhibitors [96]. Specifically, in contrast to IL-12/23 inhibitors, IL-23/39 inhibitors inhibit IL-23 without inhibiting IL-12. This is supported by data on the anti-psoriatic effects from IL-12 signaling in an imiquimod induced psoriatic-like skin inflammation model [72,97]. Currently, there are three FDA-approved IL-23/39 inhibitors: guselkumab, tildrakizumab, and risankizumab. Mirikizumab underwent phase III trials that were discontinued for administrative purposes [55,98].

Guselkumab is a monoclonal human IgG1 antibody against the p19 subunit of IL-23. Tildrakizumab is a monoclonal humanized antibody against the p19 subunit of IL-23. 

Risankizumab is a monoclonal humanized IgG1 antibody against the p19 subunit of IL-23 [55]. Mirikizumab is a monoclonal humanized IgG4 antibody against the p19 subunit of IL-23 (Table 2) (Table 5) [55,98].

Guselkumab is currently FDA-approved to treat PsA [38]. Tildrakizumab and risankizumab have completed phase II trials, and risankizumab is currently undergoing phase III trials for PsA [99].

Two multi-center phase III, double-blind, placebo- and adalimumab- controlled trials (VOYAGE 1 and VOYAGE 2) evaluated the efficacy of guselkumab in specific body regions. After 16 weeks of treatment, more patients treated with guselkumab achieved a scalp-specific-IGA score of 0 or 1 (81.8%), a hands and/or feet-PGA score of 0 or 1 (75.5%), and a fingernail-PGA score of 0 or 1 (46.7%), compared to placebo (12.4%, 14.2%, 15.2%, respectively; *p* < 0.001 for all) [100]. After 16 weeks of treatment, more patients treated with guselkumab (84.1%) achieved IGA of 0/1 compared to adalimumab (67.7%, *p* < 0.001) [101]. A multi-center phase IV randomized, double-blind, parallel-group trial assessed the efficacy of guselkumab, compared to ixekizumab in patients with moderate to severe plaque psoriasis. After 24 weeks of treatment, a similar percentage of patients treated with both guselkumab and ixekizumab achieved PASI 100 (50% and 52%, *p* = 0.41) [102]. Due to the lack of head-on-head trials between guselkumab and ustekinumab, an adjusted treatment analysis (COMPASS analysis) was done to compare the VOYAGE 1 and 2 trials for guselkumab and NAVIGATE for ustekinumab. More patients treated with guselkumab achieved PASI 90 after 16 and 40 weeks of treatment, compared to ustekinumab (Week 16: 70.4% vs. 40.6% (95% CI Week 16: 2.22–3.45); Week 40: 74.2% vs. 54.5% (95% CI Week 40: 1.89–3.13) respectively [103]. A multi-center phase III, randomized, double-blind, comparator controlled trial (ECLIPSE) evaluated the efficacy of guselkumab, compared to secukinumab, in patients with moderate to severe psoriasis. After 48 weeks of treatment, more patients treated with guselkumab (84%) achieved PASI 90 compared to secukinumab (70%, *p* < 0.0001) [104]. 

Two multi-center phase III randomized, double-blind trials (reSURFACE 1 and reSURFACE 2) evaluated the efficacy of tildrakizumab in patients with moderate-to-severe plaque psoriasis. In the reSURFACE 1 trial, after 12 weeks of treatment, more patients treated with 200 mg of tildrakizumab (65%) and 100 mg of tildrakizumab (64%) achieved PASI 75, compared to placebo (6%, *p* < 0.001 for both) [105]. In addition, more patients treated with 200 mg of tildrakizumab (59%) and 100 mg of tildrakizumab (58%) achieved a PGA of 0 or 1, compared to placebo (7%, *p* < 0.001 for both). In the reSURFACE 2 trials, after 12 weeks of treatment, more patients treated with 200 mg of tildrakizumab (66%) and 100 mg of tildrakizumab (61%) achieved PASI 75, compared etanercept (48%, *p* < 0.0001 for 200 mg vs. etanercept and *p* = 0.001 for 100 mg vs. etanercept) [105]. In addition, more patients treated with 200 mg of tildrakizumab (59%) and 100 mg of tildrakizumab (55%) achieved a PGA of 0 or 1, compared to etanercept (48%, *p* = 0.0021 for 200 mg group vs. etanercept and *p* = 0.0663 for 100 mg group vs. etanercept) [105]. 

A phase III, randomized, double-blind, active-comparator-controlled trial (IMMvent) evaluated the efficacy of risankizumab, compared to adalimumab, in patients with moderate-to-severe plaque psoriasis. After 16 weeks treatment, more patients treated with risankizumab (72%) achieved PASI 90 and an sPGA of 0 or 1 (72% and 84%, respectively), compared to patients treated with adalimumab (24.9% and 60%, *p* < 0.0001 for both) [106]. A multi-center phase III, efficacy-accessor blinded, active-comparator study evaluated risankizumab, compared to secukinumab, in patients with moderate to severe plaque psoriasis. After 16 weeks of treatment, risankizumab was not inferior to secukinumab in the number of patients who achieved PASI 90. However, more patients treated with risankizumab achieved PASI 90 (86.6%) compared to secukinumab (57.1%) after 52 weeks of treatment (*p* < 0.001) [107]. 

A multi-center phase II randomized, double blind, placebo-controlled trial (AMAF) assessed the efficacy of mirikizumab in patients with moderate-to-severe plaque psoriasis. After 16 weeks of treatment, more patients who were treated with 300 mg of mirikizumab (67%), 100 mg of mirikizumab (59%), and 30 mg of mirikizumab (29%) achieved PASI 90, compared to placebo (0%, *p* < 0.001 for 200 mg and 100 mg versus placebo *p* = 0.009 for 30 mg vs. placebo) [98].

Two network meta-analyses compared the efficacy among all IL-23/IL-39 inhibitors. A 2019 meta-analysis analyzing randomized control trials measured efficacy by percent of patients who achieved PASI 100 and ranked treatments from high to low according to their SUCRA: Risankizumab 150 mg (71%), guselkumab 100 mg (61%), tildrakizumab 100 mg (22%), and tildrakizumab 200 mg (20%) [92]. Another 2021 network meta-analysis compared the efficacy, measured by the percentage of patients who achieved PASI 90, among all IL-23/IL-39 inhibitors in psoriasis. Highest efficacy was reported in patients who received risankizumab (85.3% [95% CI: 81.4%, 88.7%]), guselkumab (78.1% [95% CI: 72.5%, 83.0%]), and ustekinumab (55.0% [95% CI: 52.7%, 57.3%]) [61]. 

The safety profiles of IL-23/IL-39 inhibitors are well described and are mild. The most commonly reported adverse events are upper respiratory tract infections and injection site reactions [2,55,56]. A 2019 meta-analysis analyzing randomized control trials measured the safety profiles of all IL-23/IL-39 inhibitors and ranked them from high to low based on short-term risk for adverse events according to their SUCRA: guselkumab 100 mg (63%), risankizumab 150 mg (67%), tildrakizumab 100 mg (89%), and tildrakizumab 200 mg (90%) [92]. Guselkumab and tildrakizumab are contraindicated in patients who have hypersensitivity to these biologics, and none of the IL-23/39 inhibitors are associated with black box warnings. (Table 5) [55]. 

### 2.4. Emerging Role of JAK Inhibitors

JAK inhibitors are an emerging class of small molecule inhibitors for the treatment of psoriasis. Currently, there are no FDA-approved JAK inhibitors in the treatment of psoriasis, however, oral tofacitinib is approved for psoriatic arthritis. Oral tofacitinib, is a JAK1 and JAK2 inhibitor and is the most studied oral JAK inhibitor in moderate to severe plaque psoriasis (Table 6) [108]. Deucravacitinib, formerly known as BMS986165, is an oral selective TYK2 inhibitor. Deucravacitinib uniquely binds to the TYK2 regulatory domain, which allows it to target the underlying pathogenesis of psoriasis through inhibition of the intracellular component of the IL-12, IL-23, and Type I interferon pathways [109,110]. 

Two replicate multicenter, phase III randomized, double-blind, placebo controlled trials (PIVOTAL 1 and PIVOTAL 2) evaluated the efficacy and safety of oral tofacitinib. After 16 weeks of treatment, more patients treated with oral tofacitinib 5 mg twice per day and oral tofacitinib 10 mg BID achieved a PGA of 0 or 1 (PIVOTAL 1: 41.9%. 59.2% and PIVOTAL 2: 46.0% and 59.1%, respectively), compared to placebo (PIVOTAL 1: 9.0% and PIVOTAL 2: 10.9%%, *p* < 0.001 for all) [111]. More patients treated with oral tofacitinib 5 mg BID and oral tofacitinib 10 mg BID achieved PASI75 (PIVOTAL 1: 39.9%. 59.2% and PIVOTAL 2: 46.0% and 59.6%, respectively), compared to placebo (PIVOTAL 1: 6.2% and PIVOTAL 2: 11.5%%, *p* < 0.001 for all). The rate of adverse events was similar across all groups, and the most commonly reported adverse effect was nasopharyngitis. Herpes zoster infections were reported in twelve patients across the tofacitinib groups in both studies [111].

Another phase III, randomized, double-blind, placebo-controlled trials evaluated the efficacy and safety of oral tofacitinib. After 16 weeks of treatment, more patients treated with oral tofacitinib 5 mg BID achieved PASI75 and a PGA of 0 or 1 (54.6%, 52.3%, respectively) and oral tofacitinib 10 mg BID (81.1%, 75.6%), compared to placebo (12.5%, 19.3%, *p* < 0.0001 for both) [112]. Patients treated with a placebo had a similar rate of adverse events as patients in the tofacitinib groups from weeks 16 to 52. The most commonly reported treatment-emergent adverse events included upper respiratory tract infections, nasopharyngitis, and hyperlipidemia [112]. 

A recently completed post-marketing study assessed the safety of tofacitinib compared to TNF-α inhibitors. Patients treated with tofacitinib experienced a higher rate of malignancy and major adverse cardiovascular events (MACE), compared to TNF-α inhibitors (malignancy: 1.13 vs. 0.77 per 100 person years; HR: 1.48; 95% CI: 1.04–2.09 and MACE: 0.98 vs. 0.73 per 100 person years; HR: 1.33; 95% CI: 0.91–1.94) [113]. Tofacitinib has several black box warnings due to the increased risk of serious infections, malignancy, and thrombosis (Table 6) [55].

A multicenter, phase II randomized, double-blind, placebo-controlled trial performed across five different countries evaluated the efficacy of deucravacitinib compared to placebo. After twelve weeks of treatment, more patients treated with 12 mg daily deucravacitinib and 6 mg twice daily achieved PASI75 (75% and 67%, respectively), compared to place (7%, *p* < 0.001 for both) [110]. Adverse events were reported in 55 to 80% of patients treated with deucravacitinib across treatment groups, compared to placebo 51% [110]. Most commonly reported adverse events included nasopharyngitis, headache, diarrhea, nausea, and upper respiratory tract infections. Treatment discontinuation due to adverse events was reported in 2 to 7% of patients treated with deucravacitinib across treatment groups, compared to placebo 4% [110]. Currently, Deucravacitinib is being investigated in multiple phase III clinical trials [109]. 

## 3. Discussion

Psoriasis is a highly prevalent chronic, systemic inflammatory skin disease with variable clinical manifestations, and plaque psoriasis is the most prevalent type. The underlying molecular pathogenesis of psoriasis can be divided into an initiation and maintenance phase. The initiation phase is mediated by keratinocyte response to injury through the release of AMPs [1]. AMPs propagate the inflammatory pathway by their effect on dendritic cells. The main AMPs involved in psoriasis pathogenesis include LL37, β-defensins, and S100 proteins [1,13]. The TNF-α/IL-23/IL-17 axis mediates the maintenance phase. IL-17 in particular works via two mechanisms: an ACT1 dependent and ACT1 independent pathway [13]. The pathogenesis of pustular psoriasis is similar to the general pathogenesis of plaque psoriasis; however, it can involve a unique mutation in *IL356RRN* [33].

The type of psoriasis, its severity, associated comorbidities, and patient preferences should be taken into consideration when choosing treatment. There are currently four classes of biologics approved for the treatment of moderate to severe plaque psoriasis. 

There are currently four FDA-approved TNF-α inhibitors: etanercept, infliximab, adalimumab, and certolizumab (and one additional one, golimumab, which is approved for psoriatic arthritis) [55]. According to data obtained from a recently completed meta-analysis, infliximab has the highest efficacy of the FDA-approved TNF-α inhibitors [61]. The adverse effects of TNF-α inhibitors are well documented. Particularly, adalimumab and infliximab have been associated with a higher risk for serious infections [64]. 

Ustekinumab is currently the only FDA-approved IL-12/IL-23 inhibitor and is a well-tolerated biologic [71]. In a Danish registry of 2161 patients, ustekinumab had the lowest risk for drug discontinuation and highest drug survival, when compared to TNF-α inhibitors [78]. 

There are currently three FDA-approved IL-17 inhibitors: secukinumab, ixekizumab, and brodalumab [80,81,82]. Bimekizumab is another IL-17 inhibitor currently undergoing phase III trials [83]. According to data from two network meta-analyses, brodalumab has the highest efficacy of the approved IL-17 drugs, but bimekizumab may be even more effective, especially for psoriatic arthritis [61,92]. According to safety data from a 2019 meta-analysis result, ixekizumab 80 mg/4 weeks has the highest short-term risk for adverse events, of the approved drugs [92]. 

There are currently three FDA-approved IL-23/IL-39 inhibitors: guselkumab, tildrakizumab, and risankizumab [55]. Mirikizumab, also an IL-23/IL-39 inhibitor, underwent phase III trials but was discontinued due to administrative purposes [98]. According to data from two network meta-analyses, risankizumab has the highest efficacy of the approved IL-23/IL-39 inhibitors [61,92]. Although IL-23/IL-39 inhibitors offer a tolerable safety profile with no black box warnings or contraindications, according to safety data from a 2019 meta-analysis, guselkumab 100 mg has the highest short-term risk for adverse events of the approved drugs [55,92]. 

Altogether, biological therapeutics offer a molecularly targeted approach for patients with plaque psoriasis. Although consideration should be taken into account for the specific contraindications and black box warnings for each medication, the overall safety profiles of biologics are favorable [114]. Generally, any specific biologic should be avoided in patients with a history of hypersensitivity to that medication [94]. Although there is scarce literature on the safety of biologics during pregnancy, secukinumab, brodalumab, risankizumab, tildrakizumab, and guselkumab have been found in breast milk in animal studies. Moreover, there is a possibility of spontaneous abortion and neonatal deaths according to animal studies, although there is no human data [115].

JAK inhibitors are also an emerging class of small molecule inhibitors, and oral tofacitinib is the most studied oral JAK inhibitor in psoriasis [108,111,112]. Although JAK inhibitors present a new small molecule inhibitor with efficacy in psoriasis, it is associated with numerous black box warnings, and careful consideration must be used when opting for these medications [55,113]. 

Biological therapies are efficacious in the treatment of psoriasis, have favorable adverse effect profiles, and improve patient quality of life. Compared to other treatment modalities, such as methotrexate and cyclosporine, the cost of treatment can be much higher [116,117,118]. However, the cost can be opaque. In the United States, insurers can contract with pharmaceutical companies and do not make the resulting costs public. In Europe, contracting occurs at the level of country-specific health plans, and they, too, may not make the price paid public. 

## 4. Materials and Methods

A review of the literature was conducted using the PubMed and Google Scholar repositories for relevant literature published between 2014–2021 utilizing the keywords: biologics, clinical manifestations, efficacy, IL-17 inhibitors, IL12/23 inhibitors, IL-23/39 inhibitors, JAK inhibitors, pathophysiology of psoriasis, pathophysiology of pustular psoriasis, psoriasis, plaque psoriasis, psoriatic arthritis, pustular psoriasis, safety profile, small molecule inhibitors, and TNF-α inhibitors. An additional Medline/PubMed database search of phase I, II, and III randomized, double-blind clinical trials published between January 2011 to November 2021 was conducted. Two authors (R. Singh and S. Koppu) read reference abstracts and included articles that focused on the molecular pathophysiology of psoriasis, associated variant pustular psoriasis, and comorbidity psoriatic arthritis, their clinical manifestations, and clinical trials, systematic, and meta-analyses of current biologic and small molecule therapeutics used to target psoriasis’ underlying pathogenesis. Articles not published in English were excluded. Publication bias was mitigated by including available results from yet-to-be-published clinical trials when applicable. A total of 119 full articles were identified. Reporting bias was mitigated by analyzing ongoing or unpublished clinical trials for the therapeutics discussed. 

## Figures and Tables

**Table 1:** Summary of TNF-α Inhibitors for Psoriasis.

TNF-α Inhibitors							
Name	Description	Stage	Route	Dosage	Adverse Effects	Contraindications	Black Box Warnings
Etanercept [2,53,54,55,56,62,66,67,68]	Recombinant fusion protein that inhibits the binding of TNF-α to TNF receptors 1 and 2	FDA approved	Subcutaneous Injection	Induction: 50 mg twice/week for first 12 weeks Maintenance: 50 mg every week	Nasopharyngitis, upper respiratory tract infections, injection site reactions	Active tuberculosis, hepatitis B, hepatitis C, advanced congestive heart failure, demyelinating diseases, sepsis (new)	Risk of serious infections and malignancy
Infliximab [2,53,54,55,56,64,66,67,69]	Chimeric monoclonal IgG1 antibody	FDA approved	Intravenous Injection	Induction: 5 mg/kg at weeks 0, 2, and 6 Maintenance: 5 mg/kg every 8 weeks	Nasopharyngitis, upper respiratory tract infections, injection site reactions	Higher doses in moderate to severe heart failure (NYHA class III or IV), current severe infection, active infection (new)	Risk of serious infection and malignancy
Adalimumab [2,53,54,55,56,63,69]	Fully human monoclonal IgG1 antibody	FDA approved	Subcutaneous Injection	Induction: 80 mg initially and 40 mg at week 1 Maintenance: 40 mg every other week	Nasopharyngitis, upper respiratory infections, injection site reactions	Active infection, sepsis, class III or IV congestive heart failure	Risk of serious infections and malignancy
Certolizumab pegol [2,53,54,55,56,57,66,67]	PEGylated TNF-alpha antibody fragment	FDA approved	Subcutaneous Injection	<90 kg: Induction: 400 mg at weeks 0, 2, and 4 Maintenance: 200 mg every 2 weeks >90 kg: 400 mg every 2 weeks	Nasopharyngitis, upper respiratory tract infections, injection site reactions	Active infection, sepsis, class III or IV congestive heart failure	Risk of serious infections and malignancy

**Table 2:** Summative Table of Drugs, Stages of Approval in the US, Japan, and Europe, Route and Dose Approved in the US [53,70].

Name	Stage in the United States	Stage in Japan	Stage in Europe
Etanercept [53,67,70]	FDA approved	Not approved	Recommended by EMA
Infliximab [53,67,70]	FDA approved	Approved	Recommended by EMA
Adalimumab [53,67,70]	FDA approved	Approved	Recommended by EMA
Certolizumab pegol [53,67,70]	FDA approved	Approved	Recommended by EMA
Ustekinumab [53,67,70]	FDA approved	Approved	Recommended by EMA
Secukinumab [53,67,70]	FDA approved	Approved	Recommended by EMA
Ixekizumab [53,67,70]	FDA approved	Approved	Recommended by EMA
Brodalumab [53,67,70]	FDA approved	Approved	Recommended by EMA
Bimekizumab [53,67,70]	Phase III	Phase III	Recommended by EMA
Guselkumab [53,67,70]	FDA approved	Approved	Recommended by EMA
Tildrakizumab [53,67,70]	FDA approved	Approved	Recommended by EMA
Risankizumab [53,67,70]	FDA approved	Approved	Recommended by EMA
Mirikizumab [53,67,70]	Phase III	Phase III	

EMA = European Medicines Agency.

**Table 3:** Summary of IL-12/23 Inhibitor.

IL-12/IL-23 Inhibitor							
Name	Description	Stage	Route	Dosage	Adverse Effects	Contraindications	Black Box Warnings
Ustekinumab [2,55,56,63,71,78,79]	Monoclonal antibody against p40 which is a subunit of IL-12 and Il-23	FDA approved	Subcutaneous Injection	<100 kg—Induction: 45 mg initially and at 4 weeks; Maintenance: 45 mg every 12 weeks >100 kg—Induction: 90 mg initially and at 4 weeks; Maintenance: 90 mg every 12 weeks	Headache, nasopharyngitis, upper respiratory tract infections, fatigue, pruritus	Active Infection	None

**Table 4:** Summary of IL-17 Inhibitors.

IL-17 Inhibitors							
Name	Description	Stage	Route	Dosage	Adverse Effects	Contraindications	Black Box Warnings
Secukinumab [55,57,67,80,81,93,94]	Monoclonal antibody that blocks IL-17A	FDA approved	Subcutaneous Injection	Induction: 300 mg at weeks 0, 1, 2, 3, 4 Maintenance: 300 mg every 4 weeks	Headache, nasopharyngitis, upper respiratory infections, mucocutaneous candidiasis	PUVA sessions, premalignancy, demyelinating disease, optic neuritis, multiple sclerosis, congestive heart failure, fever, jaundice, markedly elevated liver enzymes	None
Ixekizumab [55,57,67,80,81,93,94]	Monoclonal antibody that blocks IL-17A	FDA approved	Subcutaneous Injection	Induction: 160 mg initially and 80 mg at weeks 2, 4, 6, 8, 10, 12 Maintenance: 80 mg every 4 weeks	Headache, nasopharyngitis, upper respiratory tract infections, mucocutaneous candidiasis, injection site reactions	Hypersensitivity to ixekizumab	None
Brodalumab [55,67,95]	Monoclonal antibody that blocks IL-17 receptor type A	FDA approved	Subcutaneous Injection	Induction: 210 mg initially and weeks 1 and 2 Maintenance: 210 mg every 2 weeks	Headache, nasopharyngitis, upper respiratory tract infections, mucocutaneous candidiasis, injection site reactions	Crohn’s disease, non-resolving infection	Suicidal ideation and behavior
Bimekizumab [55,67]	Monoclonal antibody inhibiting IL-17A and IL-17F	Phase III (Clinical trials)	-	-	Nasopharyngitis, upper respiratory infections, oral candidiasis	-	-

**Table 5:** Summary of IL-23/39 Inhibitors.

IL-23/39 Inhibitors							
Name	Description	Stage	Route	Dosage	Adverse Effects	Contraindications	Black Box Warnings
Guselkumab [55,56,67]	Fully human monoclonal antibody against p19	FDA approved	Subcutaneous Injection	Induction: 100 mg initially and week 4 Maintenance: 100 mg every 12 weeks	Upper respiratory infections, nasopharyngitis, injection site reactions	Hypersensitivity to guselkumab	None
Tildrakizumab [55,56,67]	Fully human monoclonal antibody against p19	FDA approved	Subcutaneous Injection	Induction: 100 mg initially and week 4 Maintenance: 100 mg every 12 weeks	Upper respiratory infections, nasopharyngitis, injection site reactions	None Hypersensitivity to tildrakizumab	None
Risankizumab [55,56,67]	Fully human monoclonal antibody against p19	FDA approved (new)	Subcutaneous Injection	Induction: 150 mg initially and week 4 Maintenance: 150 mg every 12 weeks	Upper respiratory infections, injection site reactions, headache	None	None
Mirikizumab [55,56,67]	Monoclonal antibody against p19 (IL-23)	Phase III (Clinical trials)	-	-	Upper respiratory infections, injection-site pain, hypertension, diarrhea	-	-

**Table 6:** Summary of Oral JAK Inhibitor.

JAK Inhibitors					
Name	Description	Stage	Adverse Effects	Contraindications	Black Box Warnings
Tofacitinib [55,108,111,112,113]	JAK1 and JAK3 inhibitor	Phase III	Upper respiratory infections, nasopharyngitis,	None	Infection, malignancy, thrombosis
Deucravacitinib [109,110]	TYK2 Inhibitor	Phase III	Nasopharyngitis, headache, diarrhea, nausea, upper respiratory tract infections	-	-

## References

[1] Rendon A., Schäkel K. (2019). Psoriasis Pathogenesis and Treatment. Int. J. Mol. Sci..

[2] Armstrong A.W., Read C. (2020). Pathophysiology, Clinical Presentation, and Treatment of Psoriasis: A Review. JAMA.

[3] Schadler E.D., Ortel B., Mehlis S.L. (2019). Biologics for the primary care physician: Review and treatment of psoriasis. Disease-A-Month.

[4] Parisi R., Iskandar I.Y.K., Kontopantelis E., Augustin M., Griffiths C.E.M., Ashcroft D.M. (2020). National, regional, and worldwide epidemiology of psoriasis: Systematic analysis and modelling study. BMJ.

[5] Sawyer L.M., Malottki K., Sabry-Grant C., Yasmeen N., Wright E., Sohrt A., Borg E., Warren R.B. (2019). Assessing the relative efficacy of interleukin-17 and interleukin-23 targeted treatments for moderate-to-severe plaque psoriasis: A systematic review and network meta-analysis of PASI response. PLoS ONE.

[6] Ceccarelli M., Rullo E.V., Berretta M., Cacopardo B., Pellicanò G.F., Nunnari G., Guarneri C. (2021). New generation biologics for the treatment of psoriasis and psoriatic arthritis. State of the art and considerations about the risk of infection. Dermatol. Ther..

[7] Badri T., Kumar P., Oakley A.M. (2021). Plaque Psoriasis. StatPearls.

[8] Boehncke W.H. (2018). Systemic inflammation and cardiovascular comorbidity in psoriasis patients: Causes and consequences. Front. Immunol..

[9] Korman N.J. (2020). Management of psoriasis as a systemic disease: What is the evidence?. Br. J. Dermatol..

[10] Afonina I.S., Van Nuffel E., Beyaert R. (2021). Immune responses and therapeutic options in psoriasis. Cell. Mol. Life Sci..

[11] Petit R.G., Cano A., Ortiz A., Espina M., Prat J., Muñoz M., Severino P., Souto E.B., García M.L., Pujol M. (2021). Psoriasis: From Pathogenesis to Pharmacological and Nano-Technological-Based Therapeutics. Int. J. Mol. Sci..

[12] Sato Y., Ogawa E., Okuyama R. (2020). Role of Innate Immune Cells in Psoriasis. Int. J. Mol. Sci..

[13] Herster F., Bittner Z., Archer N.K., Dickhöfer S., Eisel D., Eigenbrod T., Knorpp T., Schneiderhan-Marra N., Löffler M.W., Kalbacher H. (2020). Neutrophil extracellular trap-associated RNA and LL37 enable self-amplifying inflammation in psoriasis. Nat. Commun..

[14] Amin M., Darji K., No D.J., Wu J.J. (2017). Review of phase III trial data on IL-23 inhibitors tildrakizumab and guselkumab for psoriasis. J. Eur. Acad. Dermatol. Venereol..

[15] Cai Y., Shen X., Ding C., Qi C., Li K., Li X., Jala V.R., Zhang H.G., Wang T., Zheng J. (2011). Pivotal role of dermal IL-17-producing γδ T cells in skin inflammation. Immunity.

[16] Matos T.R., O’Malley J.T., Lowry E.L., Hamm D., Kirsch I.R., Robins H.S., Kupper T.S., Krueger J.G., Clark R.A. (2017). Clinically resolved psoriatic lesions contain psoriasis-specific IL-17-producing αβ T cell clones. J. Clin. Investig..

[17] Teunissen M.B.M., Munneke J.M., Bernink J.H., Spuls P.I., Res P.C.M., Te Velde A., Cheuk S., Brouwer M.W.D., Menting S.P., Eidsmo L. (2014). Composition of innate lymphoid cell subsets in the human skin: Enrichment of NCR(+) ILC3 in lesional skin and blood of psoriasis patients. J. Investig. Dermatol..

[18] Boniface K., Bernard F.X., Garcia M., Gurney A.L., Lecron J.C., Morel F. (2005). IL-22 inhibits epidermal differentiation and induces proinflammatory gene expression and migration of human keratinocytes. J. Immunol..

[19] Bovenschen H.J., Gerritsen W.J., van Rens D.W.A., Seyger M.M.B., de Jong E.M.G.J., van de Kerkhof P.C.M. (2007). Explorative immunohistochemical study to evaluate the addition of a topical corticosteroid in the early phase of alefacept treatment for psoriasis. Arch. Dermatol. Res..

[20] Gilhar A., Ullmann Y., Kerner H., Assy B., Shalaginov R., Serafimovich S., Kalish R.S. (2002). Psoriasis is mediated by a cutaneous defect triggered by activated immunocytes: Induction of psoriasis by cells with natural killer receptors. J. Investig. Dermatol..

[21] Hammarén H.M., Virtanen A.T., Raivola J., Silvennoinen O. (2019). The regulation of JAKs in cytokine signaling and its breakdown in disease. Cytokine.

[22] Fardos M.I., Singh R., Perche P.O., Kelly K.A., Feldman S.R. (2021). Evaluating topical JAK inhibitors as a treatment option for atopic dermatitis. Expert Rev. Clin. Immunol..

[23] Szilveszter K.P., Németh T., Mócsai A. (2019). Tyrosine Kinases in Autoimmune and Inflammatory Skin Diseases. Front. Immunol..

[24] Raharja A., Mahil S.K., Barker J.N. (2021). Psoriasis: A brief overview. Clin. Med. (Lond.).

[25] Nogueira M., Puig L., Torres T. (2020). JAK Inhibitors for Treatment of Psoriasis: Focus on Selective TYK2 Inhibitors. Drugs.

[26] Zhou J., Luo Q., Cheng Y., Wen X., Liu J. (2021). An update on genetic basis of generalized pustular psoriasis (Review). Int. J. Mol. Med..

[27] Mahil S.K., Twelves S., Farkas K., Setta-Kaffetzi N., Burden A.D., Gach J.E., Irvine A.D., Képíró L., Mockenhaupt M., Oon H.H. (2016). AP1S3 Mutations Cause Skin Autoinflammation by Disrupting Keratinocyte Autophagy and Up-Regulating IL-36 Production. J. Investig. Dermatol..

[28] Setta-Kaffetzi N., Simpson M.A., Navarini A.A., Patel V.M., Lu H.C., Allen M.H., Duckworth M., Bachelez H., Burden A.D., Choon S.E. (2014). AP1S3 mutations are associated with pustular psoriasis and impaired Toll-like receptor 3 trafficking. Am. J. Hum. Genet..

[29] Li L., You J., Fu X., Wang Z., Sun Y., Liu H., Zhang F. (2019). Variants of CARD14 are predisposing factors for generalized pustular psoriasis (GPP) with psoriasis vulgaris but not for GPP alone in a Chinese population. Br. J. Dermatol..

[30] Sugiura K., Muto M., Akiyama M. (2014). CARD14 c.526G>C (p.Asp176His) is a significant risk factor for generalized pustular psoriasis with psoriasis vulgaris in the Japanese cohort. J. Investig. Dermatol..

[31] Sugiura K. (2014). The genetic background of generalized pustular psoriasis: IL36RN mutations and CARD14 gain-of-function variants. J. Dermatol. Sci..

[32] Wang Y., Cheng R., Lu Z., Guo Y., Yan M., Liang J., Huang P., Li M., Yao Z. (2017). Clinical profiles of pediatric patients with GPP alone and with different IL36RN genotypes. J. Dermatol. Sci..

[33] Hoegler K.M., John A.M., Handler M.Z., Schwartz R.A. (2018). Generalized pustular psoriasis: A review and update on treatment. J. Eur. Acad. Dermatol. Venereol..

[34] Qin P., Zhang Q., Chen M., Fu X., Wang C., Wang Z., Yu G., Yu Y., Li X., Sun Y. (2014). Variant analysis of CARD14 in a Chinese Han population with psoriasis vulgaris and generalized pustular psoriasis. J. Investig. Dermatol..

[35] Alinaghi F., Calov M., Kristensen L.E., Gladman D.D., Coates L.C., Jullien D., Gottlieb A.B., Gisondi P., Wu J.J., Thyssen J.P. (2019). Prevalence of psoriatic arthritis in patients with psoriasis: A systematic review and meta-analysis of observational and clinical studies. J. Am. Acad. Dermatol..

[36] Veale D.J., Fearon U. (2018). The pathogenesis of psoriatic arthritis. Lancet.

[37] Rauber S., Luber M., Weber S., Maul L., Soare A., Wohlfahrt T., Lin N.Y., DIetel K., Bozec A., Herrmann M. (2017). Resolution of inflammation by interleukin-9-producing type 2 innate lymphoid cells. Nat. Med..

[38] Kamata M., Tada Y. (2020). Efficacy and Safety of Biologics for Psoriasis and Psoriatic Arthritis and Their Impact on Comorbidities: A Literature Review. Int. J. Mol. Sci..

[39] Watanabe R., Shirai T., Namkoong H., Zhang H., Berry G.J., Wallis B.B., Schaefgen B., Harrison D.G., Tremmel J.A., Giacomini J.C. (2017). Pyruvate controls the checkpoint inhibitor PD-L1 and suppresses T cell immunity. J. Clin. Investig..

[40] Sucur A., Jajic Z., Artukovic M., Matijasevic M.I., Anic B., Flegar D., Markotic A., Kelava T., Ivcevic S., Kovacic N. (2017). Chemokine signals are crucial for enhanced homing and differentiation of circulating osteoclast progenitor cells. Arthritis Res. Ther..

[41] Tiwari V., Brent L.H. (2021). Psoriatic Arthritis.

[42] Sherlock J.P., Joyce-Shaikh B., Turner S.P., Chao C.C., Sathe M., Grein J., Gorman D.M., Bowman E.P., McClanahan T.K., Yearley J.H. (2012). IL-23 induces spondyloarthropathy by acting on ROR-γt+ CD3+CD4-CD8- entheseal resident T cells. Nat. Med..

[43] Ritchlin C.T., Colbert R.A., Gladman D.D. (2017). Psoriatic Arthritis. N. Engl. J. Med..

[44] Venken K., Jacques P., Mortier C., Labadia M.E., Decruy T., Coudenys J., Hoyt K., Wayne A.L., Hughes R., Turner M. (2019). RORγt inhibition selectively targets IL-17 producing iNKT and γδ-T cells enriched in Spondyloarthritis patients. Nat. Commun..

[45] Ljubenovic M., Lazarevic V., Golubovic M., Binic I. (2018). Integrative Approach to Psoriasis Vulgaris. Holist. Nurs. Pract..

[46] Tonini A., Gualtieri B., Panduri S., Romanelli M., Chiricozzi A. (2018). A new class of biologic agents facing the therapeutic paradigm in psoriasis: Anti-IL-23 agents. Expert Opin. Biol. Ther..

[47] Thomas L., Azad J., Takwale A. (2021). Management of nail psoriasis. Clin. Exp. Dermatol..

[48] Ji C., Wang H., Bao C., Zhang L., Ruan S., Zhang J., Gong T., Cheng B. (2021). Challenge of Nail Psoriasis: An Update Review. Clin. Rev. Allergy Immunol..

[49] Micali G., Verzì A.E., Giuffrida G., Panebianco E., Musumeci M.L., Lacarrubba F. (2019). Inverse psoriasis: From diagnosis to current treatment options. Clin. Cosmet. Investig. Dermatol..

[50] Khosravi H., Siegel M.P., Van Voorhees A.S., Merola J.F. (2017). Treatment of Inverse/Intertriginous Psoriasis: Updated Guidelines from the Medical Board of the National Psoriasis Foundation. J. Drugs Dermatol..

[51] Wang W.M., Jin H.Z. (2020). Biologics in the treatment of pustular psoriasis. Expert Opin. Drug Saf..

[52] Aslam N., Saleem H., Murtazaliev S., Quazi S.J., Khan S. (2020). FDA Approved Biologics: Can Etanercept and Ustekinumab be Considered a First-Line Systemic Therapy for Pediatric/Adolescents in Moderate to Severe Psoriasis? A Systematic Review. Cureus.

[53] Honma M., Hayashi K. (2021). Psoriasis: Recent progress in molecular-targeted therapies. J. Dermatol..

[54] Reich K., Gooderham M., Green L., Bewley A., Zhang Z., Khanskaya I., Day R.M., Goncalves J., Shah K., Piguet V. (2017). The efficacy and safety of apremilast, etanercept and placebo in patients with moderate-to-severe plaque psoriasis: 52-week results from a phase IIIb, randomized, placebo-controlled trial (LIBERATE). J. Eur. Acad. Dermatol. Venereol..

[55] Highlights of Prescribing Information. www.fda.gov/medwatch.

[56] Fda HUMIRA® (Adalimumab) Injection, for Subcutaneous Use. www.fda.gov/medwatch.

[57] Conrad C., Gilliet M. (2018). Psoriasis: From Pathogenesis to Targeted Therapies. Clin. Rev. Allergy Immunol..

[58] Bagel J., Tyring S., Rice K.C., Collier D.H., Kricorian G., Chung J., Iles J., Stolshek B.S., Kaliyaperumal A., Papp K.A. (2017). Open-label study of etanercept treatment in patients with moderate-to-severe plaque psoriasis who lost a satisfactory response to adalimumab. Br. J. Dermatol..

[59] Gottlieb A.B., Blauvelt A., Thaçi D., Leonardi C.L., Poulin Y., Drew J., Peterson L., Arendt C., Burge D., Reich K. (2018). Certolizumab pegol for the treatment of chronic plaque psoriasis: Results through 48 weeks from 2 phase 3, multicenter, randomized, double-blinded, placebo-controlled studies (CIMPASI-1 and CIMPASI-2). J. Am. Acad. Dermatol..

[60] De Vries A.C.Q., Thio H.B., de Kort W.J.A., Opmeer B.C., van der Stok H.M., de Jong E.M.G.J., Horvath B., Busschbach J.J.V., Nijsten T.E.C., Spuls P.I. (2017). A prospective randomized controlled trial comparing infliximab and etanercept in patients with moderate-to-severe chronic plaque-type psoriasis: The Psoriasis Infliximab vs. Etanercept Comparison Evaluation (PIECE) study. Br. J. Dermatol..

[61] Armstrong A.W., Soliman A.M., Betts K.A., Wang Y., Gao Y., Puig L., Augustin M. (2021). Comparative Efficacy and Relative Ranking of Biologics and Oral Therapies for Moderate-to-Severe Plaque Psoriasis: A Network Meta-analysis. Dermatol. Ther. (Heidelb).

[62] Kimball A.B., Rothman K.J., Kricorian G., Pariser D., Yamauchi P.S., Menter A., Teller C.F., Aras G., Accortt N.A., Hooper M. (2015). OBSERVE-5: Observational postmarketing safety surveillance registry of etanercept for the treatment of psoriasis final 5-year results. J. Am. Acad. Dermatol..

[63] Menter A., Thaçi D., Wu J.J., Abramovits W., Kerdel F., Arikan D., Guo D., Ganguli A., Bereswill M., Camez A. (2017). Long-Term Safety and Effectiveness of Adalimumab for Moderate to Severe Psoriasis: Results from 7-Year Interim Analysis of the ESPRIT Registry. Dermatol. Ther. (Heidelb).

[64] Kalb R.E., Fiorentino D.F., Lebwohl M.G., Toole J., Poulin Y., Cohen A.D., Goyal K., Fakharzadeh S., Calabro S., Chevrier M. (2015). Risk of Serious Infection With Biologic and Systemic Treatment of Psoriasis: Results From the Psoriasis Longitudinal Assessment and Registry (PSOLAR). JAMA Dermatol..

[65] Fiorentino D., Ho V., Lebwohl M.G., Leite L., Hopkins L., Galindo C., Goyal K., Langholff W., Fakharzadeh S., Srivastava B. (2017). Risk of malignancy with systemic psoriasis treatment in the Psoriasis Longitudinal Assessment Registry. J. Am. Acad. Dermatol..

[66] Gerriets V., Bansal P., Goyal A., Khaddour K. (2021). Tumor Necrosis Factor Inhibitors.

[67] Brownstone N.D., Hong J., Mosca M., Hadeler E., Liao W., Bhutani T., Koo J. (2021). Biologic Treatments of Psoriasis: An Update for the Clinician. Biologics.

[68] Pan A., Gerriets V. (2021). Etanercept.

[69] Fatima R., Bittar K., Aziz M. (2021). Infliximab.

[70] European Medicines Agency. https://www.ema.europa.eu/en/medicines/human/EPAR.

[71] Fda STELARA® (Ustekinumab) Injection, for Subcutaneous or Intravenous. www.fda.gov/medwatch.

[72] Rønholt K., Iversen L. (2017). Old and New Biological Therapies for Psoriasis. Int. J. Mol. Sci..

[73] European Medicines Agency. https://www.ema.europa.eu/en/medicines/human/EPAR/nepexto.

[74] Tsai T.F., Ho J.C., Song M., Szapary P., Guzzo C., Shen Y.K., Li S., Kim K.J., Kim T.Y., Choi J.H. (2011). Efficacy and safety of ustekinumab for the treatment of moderate-to-severe psoriasis: A phase III, randomized, placebo-controlled trial in Taiwanese and Korean patients (PEARL). J. Dermatol. Sci..

[75] Gordon K.B., Strober B., Lebwohl M., Augustin M., Blauvelt A., Poulin Y., Papp K.A., Sofen H., Puig L., Foley P. (2018). Efficacy and safety of risankizumab in moderate-to-severe plaque psoriasis (UltIMMa-1 and UltIMMa-2): Results from two double-blind, randomised, placebo-controlled and ustekinumab-controlled phase 3 trials. Lancet.

[76] Lebwohl M., Strober B., Menter A., Gordon K., Weglowska J., Puig L., Papp K., Spelman L., Toth D., Kerdel F. (2015). Phase 3 studies comparing brodalumab with ustekinumab in psoriasis. N. Engl. J. Med..

[77] Bagel J., Blauvelt A., Nia J., Hashim P., Patekar M., de Vera A., Ahmad K., Paguet B., Xia S., Muscianisi E. (2021). Secukinumab maintains superiority over ustekinumab in clearing skin and improving quality of life in patients with moderate to severe plaque psoriasis: 52-week results from a double-blind phase 3b trial (CLARITY). J. Eur. Acad. Dermatol. Venereol..

[78] Egeberg A., Ottosen M.B., Gniadecki R., Broesby-Olsen S., Dam T.N., Bryld L.E., Rasmussen M.K., Skov L. (2018). Safety, efficacy and drug survival of biologics and biosimilars for moderate-to-severe plaque psoriasis. Br. J. Dermatol..

[79] Colquhoun M., Kemp A.K. (2021). Ustekinumab.

[80] Krueger J.G., Wharton K.A., Schlitt T., Suprun M., Torene R.I., Jiang X., Wang C.Q., Fuentes-Duculan J., Hartmann N., Peters T. (2019). IL-17A inhibition by secukinumab induces early clinical, histopathologic, and molecular resolution of psoriasis. J. Allergy Clin. Immunol..

[81] Fda TALTZ (Ixekizumab) Injection, for Subcutaneous Use. www.fda.gov/medwatch.

[82] Griffiths C.E.M., Reich K., Lebwohl M., Van De Kerkhof P., Paul C., Menter A., Cameron G.S., Erickson J., Zhang L., Secrest R.J. (2015). Comparison of ixekizumab with etanercept or placebo in moderate-to-severe psoriasis (UNCOVER-2 and UNCOVER-3): Results from two phase 3 randomised trials. Lancet.

[83] Gordon K.B., Foley P., Krueger J.G., Pinter A., Reich K., Vender R., Vanvoorden V., Madden C., White K., Cioffi C. (2021). Bimekizumab efficacy and safety in moderate to severe plaque psoriasis (BE READY): A multicentre, double-blind, placebo-controlled, randomised withdrawal phase 3 trial. Lancet.

[84] Adams R., Maroof A., Baker T., Lawson A.D.G., Oliver R., Paveley R., Rapecki S., Shaw S., Vajjah P., West S. (2020). Bimekizumab, a Novel Humanized IgG1 Antibody That Neutralizes Both IL-17A and IL-17F. Front. Immunol..

[85] Ali Z., Matthews R., Al-Janabi A., Warren R.B. (2021). Bimekizumab: A dual IL-17A and IL-17F inhibitor for the treatment of psoriasis and psoriatic arthritis. Expert Rev. Clin. Immunol..

[86] Mease P.J., Helliwell P.S., Hjuler K.F., Raymond K., Mcinnes I. (2021). Brodalumab in psoriatic arthritis: Results from the randomised phase III AMVISION-1 and AMVISION-2 trials. Ann. Rheum. Dis..

[87] Blauvelt A., Reich K., Tsai T.F., Tyring S., Vanaclocha F., Kingo K., Ziv M., Pinter A., Vender R., Hugot S. (2017). Secukinumab is superior to ustekinumab in clearing skin of subjects with moderate-to-severe plaque psoriasis up to 1 year: Results from the CLEAR study. J. Am. Acad. Dermatol..

[88] Papp K.A., Reich K., Paul C., Blauvelt A., Baran W., Bolduc C., Toth D., Langley R.G., Cather J., Gottlieb A.B. (2016). A prospective phase III, randomized, double-blind, placebo-controlled study of brodalumab in patients with moderate-to-severe plaque psoriasis. Br. J. Dermatol..

[89] Reich K., Papp K.A., Blauvelt A., Langley R.G., Armstrong A., Warren R.B., Gordon K.B., Merola J.F., Okubo Y., Madden C. (2021). Bimekizumab versus ustekinumab for the treatment of moderate to severe plaque psoriasis (BE VIVID): Efficacy and safety from a 52-week, multicentre, double-blind, active comparator and placebo controlled phase 3 trial. Lancet.

[90] Warren R.B., Blauvelt A., Bagel J., Papp K.A., Yamauchi P., Armstrong A., Langley R.G., Vanvoorden V., De Cuyper D., Cioffi C. (2021). Bimekizumab versus Adalimumab in Plaque Psoriasis. N. Engl. J. Med..

[91] Reich K., Warren R.B., Lebwohl M., Gooderham M., Strober B., Langley R.G., Paul C., De Cuyper D., Vanvoorden V., Madden C. (2021). Bimekizumab versus Secukinumab in Plaque Psoriasis. N. Engl. J. Med..

[92] Bai F., Li G.G., Liu Q., Niu X., Li R., Ma H. (2019). Short-Term Efficacy and Safety of IL-17, IL-12/23, and IL-23 Inhibitors Brodalumab, Secukinumab, Ixekizumab, Ustekinumab, Guselkumab, Tildrakizumab, and Risankizumab for the Treatment of Moderate to Severe Plaque Psoriasis: A Systematic Review and Network Meta-Analysis of Randomized Controlled Trials. J. Immunol. Res..

[93] Preuss C.V., Quick J. (2021). Ixekizumab.

[94] Aboobacker S., Kurn H., Al Aboud A.M. (2021). Secukinumab.

[95] Golbari N.M., Basehore B.M., Zito P.M. (2021). Brodalumab.

[96] Lu Z., Xu K., Wang X., Li Y., Li M. (2020). Interleukin 39: A new member of interleukin 12 family. Cent. J. Immunol..

[97] Kulig P., Musiol S., Freiberger S.N., Schreiner B., Gyülveszi G., Russo G., Pantelyushin S., Kishihara K., Alessandrini F., Kündig T. (2016). IL-12 protects from psoriasiform skin inflammation. Nat. Commun..

[98] Reich K., Rich P., Maari C., Bissonnette R., Leonardi C., Menter A., Igarashi A., Klekotka P., Patel D., Li J. (2019). Efficacy and safety of mirikizumab (LY3074828) in the treatment of moderate-to-severe plaque psoriasis: Results from a randomized phase II study. Br. J. Dermatol..

[99] Yang K., Oak A.S.W., Elewski B.E. (2021). Use of IL-23 Inhibitors for the Treatment of Plaque Psoriasis and Psoriatic Arthritis: A Comprehensive Review. Am. J. Clin. Dermatol..

[100] Foley P., Gordon K., Griffiths C.E.M., Wasfi Y., Randazzo B., Song M., Li S., Shen Y.K., Blauvelt A. (2018). Efficacy of guselkumab compared with adalimumab and placebo for psoriasis in specific body regions a secondary analysis of 2 randomized clinical trials. JAMA Dermatol..

[101] Reich K., Armstrong A.W., Foley P., Song M., Wasfi Y., Randazzo B., Li S., Shen Y.K., Gordon K.B. (2017). Efficacy and safety of guselkumab, an anti-interleukin-23 monoclonal antibody, compared with adalimumab for the treatment of patients with moderate to severe psoriasis with randomized withdrawal and retreatment: Results from the phase III, double-blind, placebo- and active comparator-controlled VOYAGE 2 trial. J. Am. Acad. Dermatol..

[102] Blauvelt A., Leonardi C., Elewski B., Crowley J.J., Guenther L.C., Gooderham M., Langley R.G., Vender R., Pinter A., Griffiths C.E.M. (2021). IXORA-R Study Group. A head-to-head comparison of ixekizumab vs. guselkumab in patients with moderate-to-severe plaque psoriasis: 24-week efficacy and safety results from a randomized, double-blinded trial. Br. J. Dermatol..

[103] Diels J., Thilakarathne P., Cameron C., McElligott S., Schubert A., Puig L. (2020). Adjusted treatment COMPArisons between guSelkumab and uStekinumab for treatment of moderate-to-severe plaque psoriasis: The COMPASS analysis. Br. J. Dermatol..

[104] Reich K., Armstrong A.W., Langley R.G., Flavin S., Randazzo B., Li S., Hsu M.-C., Branigan P., Blauvelt A. (2019). Guselkumab versus secukinumab for the treatment of moderate-to-severe psoriasis (ECLIPSE): Results from a phase 3, randomised controlled trial. Lancet.

[105] Reich K., Papp K.A., Blauvelt A., Tyring S.K., Sinclair R., Thaçi D., Nograles K., Mehta A., Cichanowitz N., Li Q. (2017). Tildrakizumab versus placebo or etanercept for chronic plaque psoriasis (reSURFACE 1 and reSURFACE 2): Results from two randomised controlled, phase 3 trials. Lancet.

[106] Reich K., Gooderham M., Thaçi D., Crowley J.J., Ryan C., Krueger J.G., Tsai T.F., Flack M., Gu Y., Williams D.A. (2019). Risankizumab compared with adalimumab in patients with moderate-to-severe plaque psoriasis (IMMvent): A randomised, double-blind, active-comparator-controlled phase 3 trial. Lancet.

[107] Warren R.B., Blauvelt A., Poulin Y., Beeck S., Kelly M., Wu T., Geng Z., Paul C. (2021). Efficacy and safety of risankizumab vs. secukinumab in patients with moderate-to-severe plaque psoriasis (IMMerge): Results from a phase III, randomized, open-label, efficacy-assessor-blinded clinical trial. Br. J. Dermatol..

[108] Słuczanowska-Głąbowska S., Ziegler-Krawczyk A., Szumilas K., Pawlik A. (2021). Role of Janus Kinase Inhibitors in Therapy of Psoriasis. J. Clin. Med..

[109] Bellinato F., Gisondi P., Girolomoni G. (2021). Latest Advances for the Treatment of Chronic Plaque Psoriasis with Biologics and Oral Small Molecules. Biologics.

[110] Papp K., Gordon K., Thaçi D., Morita A., Gooderham M., Foley P., Girgis I.G., Kundu S., Banerjee S. (2018). Phase 2 Trial of Selective Tyrosine Kinase 2 Inhibition in Psoriasis. N. Engl. J. Med..

[111] Papp K.A., Menter M.A., Abe M., Elewski B., Feldman S.R., Gottlieb A.B., Langley R., Luger T., Thaci D., Buonanno M. (2015). OPT Pivotal 1 and OPT Pivotal 2 investigators. Tofacitinib, an oral Janus kinase inhibitor, for the treatment of chronic plaque psoriasis: Results from two randomized, placebo-controlled, phase III trials. Br. J. Dermatol..

[112] Zhang J.Z., Tsai T.F., Lee M.G., Zheng M., Wang G., Jin H.Z., Gu J., Li R.Y., Liu Q.Z., Chen J. (2017). The efficacy and safety of tofacitinib in Asian patients with moderate to severe chronic plaque psoriasis: A Phase 3, randomized, double-blind, placebo-controlled study. J. Dermatol. Sci..

[113] Safety Study of Tofacitinib Versus Tumor Necrosis Factor (TNF) Inhibitor in Subjects with Rheumatoid Arthritis—Study Results ClinicalTrials.gov. https://clinicaltrials.gov/ct2/show/NCT02092467.

[114] Sbidian E., Chaimani A., Garcia-Doval I., Doney L., Dressler C., Hua C., Hughes C., Naldi L., Afach S., Le Cleach L. (2017). Systemic pharmacological treatments for chronic plaque psoriasis: A network meta-analysis. Cochrane Database Syst. Rev..

[115] Yeung J., Gooderham M.J., Grewal P., Hong C.-H., Lansang P., Papp K.A., Poulin Y., Turchin I., Vender R. (2020). Management of Plaque Psoriasis With Biologic Therapies in Women of Child-Bearing Potential Consensus Paper. J. Cutan. Med. Surg..

[116] Poulin Y., Langley R., Teixeira H.D., Martel M.J., Cheung S. (2009). Biologics in the treatment of psoriasis: Clinical and economic overview. J. Cutan. Med. Surg..

[117] Stein K.R., Pearce D.J., Feldman S.R. (2005). The impact of biologics on the quality of life of psoriasis patients and the economics of psoriasis care. Semin. Cutan. Med. Surg..

[118] Küster D., Nast A., Gerdes S., Weberschock T., Wozel G., Gutknecht M., Schmitt J. (2016). Cost-effectiveness of systemic treatments for moderate-to-severe psoriasis in the German health care setting. Arch. Dermatol. Res..

